# Di-(2-ethylhexyl) phthalate induces precocious puberty in adolescent female rats 

**DOI:** 10.22038/IJBMS.2018.28489.6905

**Published:** 2018-08

**Authors:** Te Liu, Yuzhuo Wang, Modi Yang, Pu Shao, Lian Duan, Meng Li, Mingji Zhu, Jie Yang, Jinlan Jiang

**Affiliations:** 1 Scientific Research Center, China-Japan Union Hospital of Jilin University, Changchun, 130033, China; 2 Department of Orthodontics, School and Hospital of Stomatology, Jilin University, Changchun, 130021, China; 3 Department of Orthopedics, China-Japan Union Hospital of Jilin University, Changchun 130033, China; 4 Scientific Research Center, China-Japan Union Hospital of Jilin University, Changchun, 130033, China; 5 Department of Dermatological, China-Japan Union Hospital of Jilin University, Changchun, 130033, China; 6 Department of Endocrinology, China-Japan Union Hospital of Jilin University, Changchun, 130033, China

**Keywords:** Di-(2-ethylhexyl) phthalate, Female, Hypothalamus, Precocious puberty, Reproductive

## Abstract

**Objective(s):**

Nowadays, Di-(2-ethylhexyl) phthalate (DEHP) is widely used in different kinds of commercial products as a plasticizer. Previous studies have revealed that exposures to DEHP could be associated with precocious puberty in teenagers, but the exact mechanism is yet to be known.

**Materials and Methods:**

In this study, 48 prepubertal Wistar female rats were randomly apportioned into 4 groups and orally treated with 0, 250, 500, and 1000 mg/kg/d DEHP from postnatal day 21 up to 4 weeks. Subsequently, we examined the indicators related to the initiation of sexual development.

**Results:**

DEHP was able to shorten the vaginal opening time and prolong the estrous cycles of female rats. IGF-1 expression was significantly upregulated by 1000 mg/kg/d DEHP in the hypothalamus, and the hypothalamic, as well as serum levels of GH, were also upregulated by DEHP. It also caused decrements in serum levels of FSH, LH, and T and the increment in level of progesterone. Meanwhile, DEHP was able to exert its effect on the mRNA and protein expression levels of Kiss-1, GPR54, and GnRH in the hypothalamus in pubertal female rats.

**Conclusion:**

These findings are revealing that DEHP exposure more likely causes imbalances of hypothalamus functioning in pubertal female rats and thus induces precautious puberty in these animals.

In recent years, the number of children with precocious puberty especially amongst girls has dramatically increased (1). Epidemiological investigations showed that the age of puberty development in children around the world continues to advance, and the girls’ average age of menarche in Australia, Europe, and the United States was lowered 3 years compared to 2010 (2-5). Some research has found that exposure to environmental endocrine disruptors is related to precocious puberty of children (6-8).

Di-(2-ethylhexyl) phthalate (DEHP) is one of the environmental endocrine disruptors which are used in a large variety of industrial and consumer applications, such as medical supplies, toys, packaging bags, cleaning agents, cosmetics, interior decoration, and decoration materials (9-11).  It is to be regretted that DEHP may leach slowly from these plastic products into foods, beverages, and even directly into body fluids (10, 12), and it is also detected in the atmosphere, water, and soil (13). DEHP has become a common concern as its effect can be amplified through biological concentration and enters the human body in high concentration (11).

DEHP and its metabolite, mono- (2-ethylhexyl) phthalate (MEHP), have a variety of toxic effects on the human body, which is the most serious damage to the reproductive endocrine system (14). DEHP can cause diseases such as sexual precocity, infertility, abortion, and uterine bleeding and thus arouse concerns from the society and scholars. It can cause abortion of female animals, increased ovarian weight, and follicle developmental disorders. Gonadal synthesis of estradiol and gonadotropin receptor gene expression decreased pituitary gonadotropin, up-regulated genes (15, 16), and lead to infertility, sexual precocity, and uterine bleeding (17, 18). 

Hitherto, previous research has focused on the reproductive toxicity of adult females’ exposure to DEHP. However, limited information is known on the link between DEHP exposure and precocious puberty in pubertal female rats. With the development of society and economy, precocious puberty in girls has been got more and more attention. Animal reproduction is regulated by kinds of factors both inside and outside the body, and the hypothalamus-pituitary-gonadal axis plays an important regulatory role in it. Kisspeptin belongs to a family of peptides, encoded by the Kiss-1 gene, mostly located in the arcuate nucleus (ARC) and anteroventral periventricular (AVPV) region in rat brain (19). The Kiss-1 gene of hypothalamus binds and activates the G protein-coupled receptor 54 (GPR54) to regulate the synthesis and secretion of GnRH, and then initiate and regulate the hypothalamic-pituitary-ovarian axis (HPOA) to enter puberty (20-22). 

Before puberty, exposure to exogenous estrogen or environmental endocrine disruptors can cause abnormal development (23), and disruptive factors may affect the neural system in puberty (24). Epidemiology surveys have found that sexual precocity and exposure to DEHP are associated (6, 25). Thus, the molecular mechanism by which the initiation of puberty is influenced by the DEHP treatment is still unknown. In this study, we will observe the effects of DEHP exposure on the related indexes of precocious puberty in female rats, and find the regulatory role of HPOA in female precocious puberty induced by DEHP. It may provide new targets for the prevention and treatment of precocious puberty in girls.

## Materials and Methods

***Animal care and DEHP exposure***

Young Wistar female rats (n=48, 15 days old) were allowed at least a 7-day acclimatization-period and observed for signs of illness before starting experimental procedures. Rats were housed in a 12:12 hr dark/light cycle in a thermometric room (22 ± 2 ^°^C). Animals were randomly apportioned into 4 treatment groups (n = 12, each): 0, 250 (1/120 LD_50_), 500 (1/60 LD_50_), and 1000 (1/30 LD_50_) mg/kg/d DEHP (Sinopharm Chemical Reagent, purity >99%) in 0.5 mL corn oil/100 g body weight for up to 4 weeks. In order to have a better observation on the toxic effects and mechanisms, the DEHP doses were set from the LD_50_ (30 g/kg) in rats (26) and the human exposure dose (5 mg/kg/d) (27). The vaginal opening time and estrous cycle were observed in female rats every day.

***Tissue sampling***

Rats were submitted and killed by decapitation. Blood was collected and the serum was separated and stored at -80 ^°^C until the hormone levels assay. The hypothalamus of each rat was also dissected out. Three hypothalami per group were used for histopathological and immunohistochemistry analysis. For the remaining rats in the same group, the hypothalami were stored at -80 ^°^C and used for Western blotting and RT-qPCR analysis.

***Quantification of GH and IGF-1***

Growth hormone (GH) and Insulin growth factor 1 (IGF-1) in the hypothalamus and serum were detected using enzyme-linked immunosorbent assay kits (R&D systems, USA). Each sample was mixed with 10 µl GH, IGF-1 antibody, and 50 µl horseradish peroxidase-labeled streptavidin, and then incubated at 37 ^°^C for 30 min. The well plates were rinsed five times with washing lotion and hatched with chromogen solution from the kit (50 µl A and 50 µl B) for 10 min at 37 ^°^C away from light. The reaction was stopped with 50 µl of stop solution, and the absorbance was read at 450 nm within 10 min. The termination solution was added to stop the reaction, and the absorbance was measured at 450 nm within 10 min.

***Radioimmunoassay***

Follicle-stimulating hormone (FSH), luteinizing hormone (LH), estradiol (E_2_), progesterone (P), and testosterone (T) of serum were assayed by radioimmunoassay using a kit (Union Medical & Pharmaceutical Technology, China), and ^125^I as a tracer. The results were expressed as IU/L, ng/mL, or pg/mL.

***Immunohistochemistry***

Samples were placed in 3.7% formaldehyde solution for 24 hr and then rehydrated through graded ethanols. The fixed hypothalamus was paraffin-embedded, sectioned and mounted on glass slides. The specimens were incubated at room temperature for 10 min, fixed in formaldehyde for 10 min, and then boiled in citric acid solution for 5 min. The sections were followed by the primary antibody (1:100, Bioss Biotechnology Company, China) overnight at 4 ^°^C. The slides were incubated with the peroxidase-conjugated anti-rabbit secondary antibody (1:100, Bioss Biotechnology Company, China) for 30 min at room temperature. The dyeing was envisioned using a 3, 3’-diaminobenzidine (DAB) (Bioss Biotechnology Company) kit. The slides were observed under a light microscope and photographed. Kiss-1, GPR54, and GnRH expression of immunohistochemical staining was performed using the integrated optical density (IOD), which showed the change of positive material optical density and area by the Image-Pro Plus 6.0 software. 

***RNA extraction***

Total RNA was extracted from the hypothalamus using Trizol reagent (Takara, Japan). The quantity and the integrality of total RNA were determined by an ultraviolet spectrophotometer and estimated by formaldehyde modified agarose gel electrophoresis.

**Figure 1:**
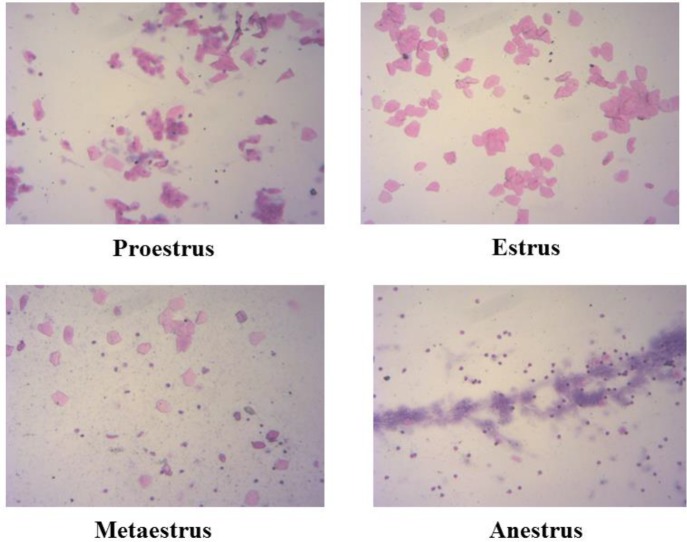
Morphological changes of vaginal epithelial cells in pubertal female rats (×100)

**Figure 2:**
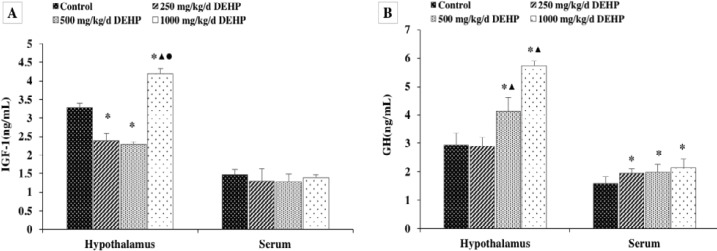
Effect of DEHP on GH and IGF-1 levels in pubertal female rats (n=12). GH and IGF-1 levels in the hypothalamus were expressed as the mean value ± standard error. *Significant difference compared with controls (*P*<0.05); ^▲^Significant difference compared with 250 mg/kg/d (*P*<0.05); ^●^Significant difference compared with 500 mg/kg/d (*P*<0.05)

**Table 1:** Gene-specific forward and reverse primer sequence

Target gene	Direction	Sequence
*Kiss-1*	Forward	CACCTGTGGTGAACCCTGAA
	Reverse	TTTGCCAGGCATTAACGAGT
*GPR54*	Forward	GCGGCCACAGATGTCACTTT
	Reverse	AGGTGGGCAGCGGATAGAG
*GnRH*	Forward	TCCAGCCAGCACTGGTCCTA
	Reverse	GGGTTCTGCCATTTGATCCTC
*β-actin*	Forward	CACCCGCGAGTACAACCTTC
	Reverse	CCCATACCCACCATCACACC

**Table 2:** Effect of DEHP on vaginal opening time in pubertal female rats $\bar{x}\pm s$ (day)

Groups	n	Vaginal opening time
Control 250mg/kg/d 500mg/kg/d 1000mg/kg/d	12 12 12 12	35.50±3.27 34.30±3.80 34.00±4.06 29.58±3.85*▲●

* *P*<0.05, vs control group;

▲ *P*<0.05, vs 250 mg/kg/d group;

● *P*<0.05, vs 500 mg/kg/d group

**Table 3:** Effect of DEHP on estrous cycle in pubertal female rats $\bar{x}\pm s$ (day)

Groups	n	Proestrus	Estrus	Metaestrus	Anestrus	Estrous Cycle
Control	12	16.80±6.57	14.80±3.90	12.00±0.00	38.40±5.37	86.80±13.97
250mg/kg/d	12	20.80±5.22	16.40±4.98	13.20±2.68	44.40± 5.37	94.80±11.10
500mg/kg/d	12	25.60±6.07	20.00±3.74	20.80±3.03	49.60±6.07*	116.00±17.20*
1000mg/kg/d	12	23.20±1.79	18.20±5.76	26.22±13.76	60.60±7.40*▲●	128.20±23.09*▲

* *P*<0.05, vs control group;

▲ *P*<0.05, vs 250 mg/kg/d group;

● *P*<0.05, vs 500 mg/kg/d group

**Figure 3:**
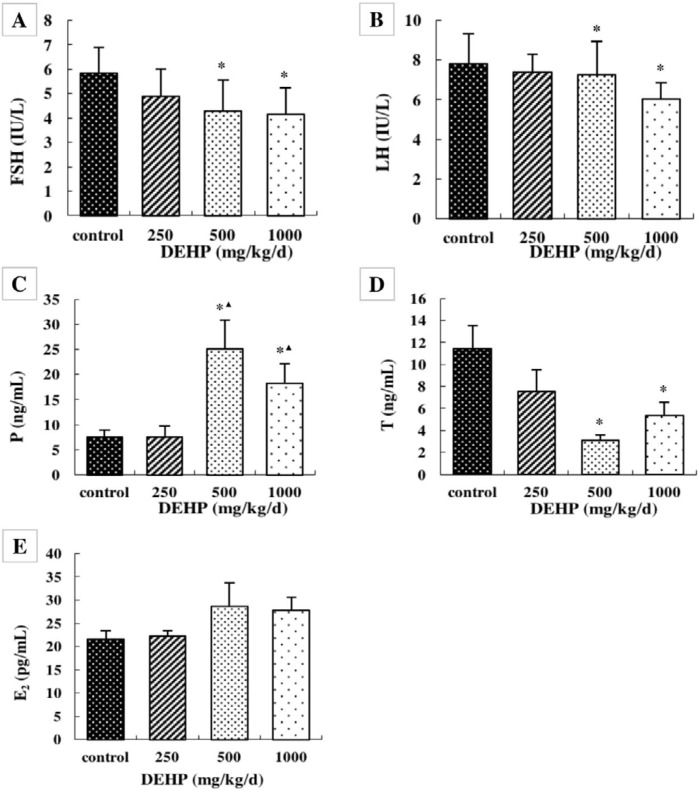
Effects of DEHP on blood serum levels of (A) FSH, (B) LH, (C) P4, (D) T, and (E) E2; n=12. Serum levels were expressed as the mean value ± standard error. *Significant difference compared with controls (*P*<0.05); ^▲^Significant difference compared with 250 mg/kg/d (*P*<0.05); ^●^Significant difference compared with 500 mg/kg/d (*P*<0.05)

**Figure 4:**
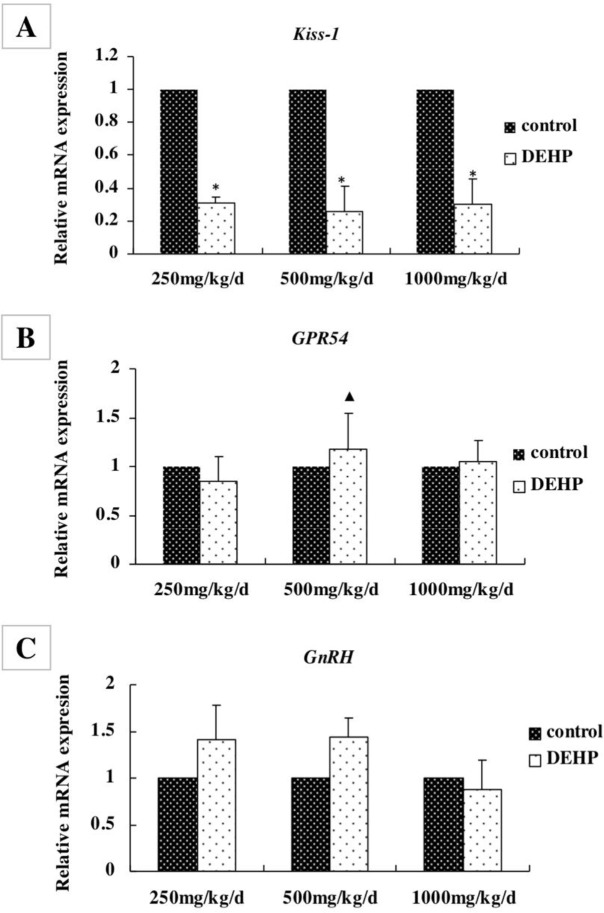
Effects of DEHP exposure on the levels of (A) Kiss-1, (B) GPR54 and (C) GnRH mRNA in the hypothalamus; n=12. The mRNA levels were expressed as the mean value ± standard error. *Significant difference compared with controls (*P*<0.05);^▲^Significant difference compared with 250 mg/kg/d (*P*<0.05); ^●^Significant difference compared with 500 mg/kg/d (*P*<0.05)

**Figure 5:**
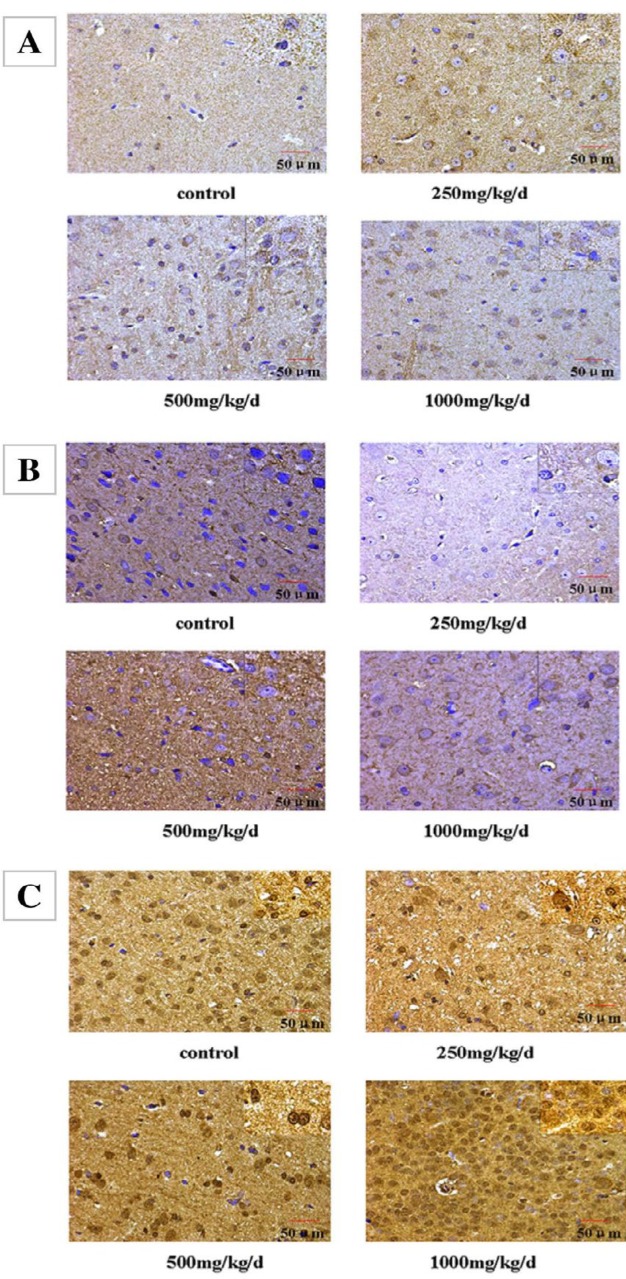
Immunohistochemical staining of (A) Kiss-1, (B) GPR54, and (C) GnRH expression in the hypothalamus of pubertal female rats (×400 and ×100)

**Figure 6:**
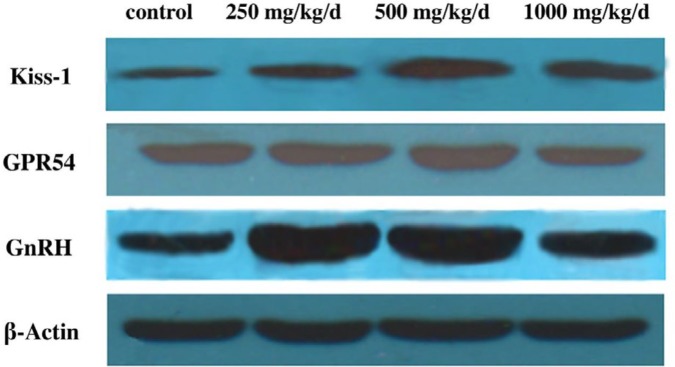
Protein bands of Kiss-1, GPR54, and GnRH expression in the hypothalamus of pubertal female rats

**Figure 7:**
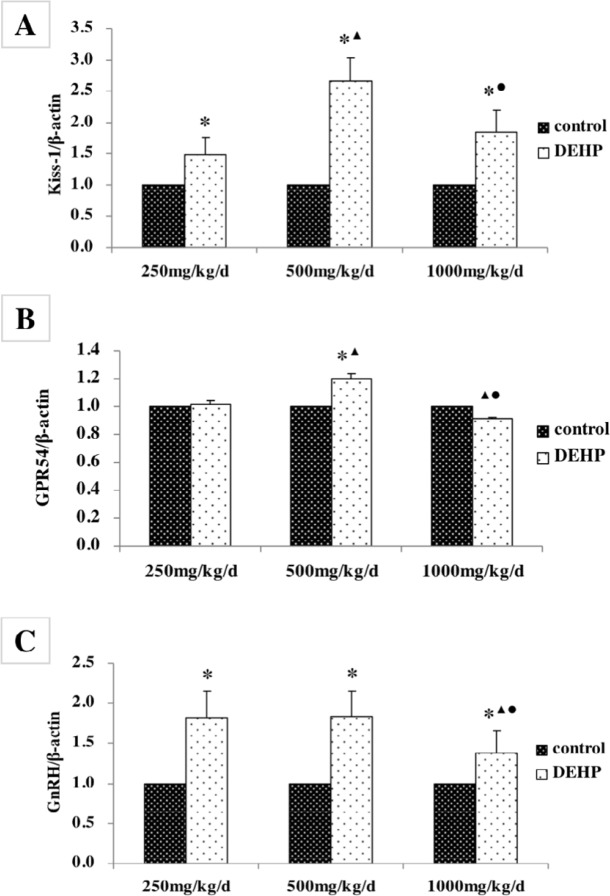
Effects of DEHP exposure on the protein levels of (A) Kiss-1, (B) GPR54, and (C) GnRH in the hypothalamus; n=12. The protein levels were expressed as the mean value ± standard error. *Significant difference compared with controls (*P*<0.05); ^▲^Significant difference compared with 250 mg/kg/d (*P* < 0.05); ^●^Significant difference compared with 500 mg/kg/d (*P*<0.05)

**Table 4:** Effects of DEHP on IOD/area of Kiss-1, GPR54, and GnRH staining in the hypothalamus

Groups	n	IOD/area (×10^-2^)		
		Kiss-1	GPR54	GnRH
Control	12	13.433±0.641	12.409±0.943	15.322±0.853
250mg/kg/d	12	16.212±0.652*	12.437±0.615	17.081±0.728*
500mg/kg/d	12	17.310±1.405*	16.387±1.323*▲	16.742±0.537*
1000mg/kg/d	12	16.719±0.221*	13.782±1.238●	16.492±0.521*

* *P*<0.05 vs control group;

▲ *P*<0.05 vs 250 mg/kg/d group;

● *P*<0.05 vs 500 mg/kg/d group

***Real-time quantitative reverse transcription-PCR***

Real-time *quantitative* RT-PCR was used to measure the expression of target genes, including β-actin, using Stratagene MX3000p (Japan). The reverse transcription was performed with 500 ng total RNA in a 10-μl reaction, and 1 μl of cDNA was then used for a 10-μl PCR reaction mixture containing an optimal concentration of primers and SYBR-Green Supermix (Takara, Japan). β-actin was used as an internal control. The primers are listed in Table 1. 

***Western blotting***

The levels of key proteins in the hypothalamus were measured by Western blotting to confirm the effects of DEHP on precocious puberty of female rats. Tissues were homogenized in RIPA lysis buffer with protease inhibitors (Beyotime, China), and then centrifuged at 12,000 g for 5 min at 4^ °^C. Protein concentration was mensurated using BCA protein assay (Beyotime, China). Equal amounts of protein from each sample were mixed with SDS sample buffer (Beyotime, China). Samples were submitted to standard SDS-PAGE and Western blotting. The primary antibodies used were: rabbit polyclonal anti-Kiss-1 antibody (1:500, Abcam, USA), rabbit polyclonal anti-GPR54 antibody (1:500, Santa Cruz, USA), mouse polyclonal anti-GnRH antibody (1:200, Abcam, USA), and rabbit polyclonal anti-β-actin antibody (1:1000, Abcam, USA), and the marker protein was used for regulation. Samples were then incubated with fluorescence secondary antibodies (1:2000, Proteintech, USA) for 1 hr at room temperature. The immunolabeling was observed by Odyssey infrared imaging system (LI-COR, USA) and the relative optical densities of bands were detected using the Image J software (USA). Western blotting analysis was performed repetitively for each sample and its average level was calculated for comparison. 

***Statistical analysis***

Statistical evaluations were analyzed for normal distribution and independence using the SPSS 22.0 statistical software. Differences between the treatment and control groups were tested by analysis of variance (ANOVA) followed by the Tukey’s honest significance difference test. The values were expressed as means ± standard error of the means. *P*<0.05 was considered significant. 

## Results

***Effect of DEHP on vaginal opening time in pubertal female rats***

In Table 2, the results showed that the vaginal opening time of the 1000 mg/kg/d dose group was significantly earlier than that of the control and other groups (*P*<0.05).

***Effect of DEHP on estrous cycle in pubertal female rats***

According to the morphological changes of vaginal epithelial cells, and the estrous cycle of pubertal female rats in four groups were analyzed (Figure 1 and Table 3). The results showed that the estrous cycles of 500 and 1000 mg/kg/d dose groups were significantly longer than that of the control group (*P*<0.05), and the estrous cycle of the 1000 mg/kg/d dose group was significantly longer than that of the 250 mg/kg/d dose group (*P*<0.05).

***Effect of DEHP on the levels of IGF-1 and GH***

The effect of DEHP on IGF-1 and GH levels in the hypothalamus and serum was also examined. Here we found that the level of IGF-1 in the hypothalamus was significantly lower in the rats treated with 250 and 500 mg/kg/d DEHP compared with the controls, and the levels in rats treated with 1000 mg/kg/d DEHP were significantly higher than in the rats in all other groups, while there was no difference in the serum level of IGF-1 (Figure 2A, *P*>0.05). 

As shown in Figure 2B, the GH levels were significantly higher in both hypothalamus and serum of rats treated with DEHP compared with the control rats (*P*<0.05). 

***Effect of DEHP on serum sex hormone levels***

The serum levels of FSH, LH, and testosterone were significantly lower in the rats treated with 500 and 1000 mg/kg/d DEHP compared with the rats in control, while the serum P was higher in the rats treated with 500 and 1000 mg/kg/d DEHP compared with the rats in the control and 250 mg/kg/d DEHP groups (Figure 4, *P*<0.05). There was no obvious change in the serum level of E_2_ after treatment with any concentration of DEHP (Figure 3, *P*>0.05).

***Gene expression levels in the hypothalamus***

The mRNA levels of Kiss-1, GPR54, and GnRH in the hypothalamus were measured by real-time RT-PCR (Figure 4). The level of Kiss-1 was significantly lower in rats treated with 250, 500, and 1000 mg/kg/d DEHP, compared with the control rats (Figure 4A, *P*<0.05). The level of GPR54 was higher in the rats treated with 500 mg/kg/d DEHP compared with the rats in the 250 mg/kg/d DEHP groups (Figure 4B, *P*<0.05). There was no significant change in the mRNA level of GnRH after treatment with any concentration of DEHP (Figure 4C, *P*>0.05).

***Immunohistochemical staining in the hypothalamus***

Kiss-1, GPR54, and GnRH localized in the hypothalamus were examined by immunohistochemistry (Figure 5). Our results showed that positive staining for Kiss-1 was found in the cytoplasm and membranes of neuroepithelial cells and neurons (Figure 5A). The IOD/areas of Kiss-1 in the hypothalamus were significantly higher in rats treated with 250, 500, and 1000 mg/kg/d compared with the control rats (*P*<0.05; Table 4). These data indicate that treatment with DEHP was associated with elevated Kiss-1 levels in the hypothalamus. 

The positive staining for GPR54 was observed in the cytoplasm and membranes of neuroepithelial cells and the neuronal cell membrane (Figure 5B). The IOD/area of GPR54 was significantly higher only in those treated with 500 mg/kg/d DEHP, compared with other groups (*P*<0.05, Table 4).

As shown in Figure 5C, the positive staining for GnRH was observed in the cytoplasm, membrane, and nucleus of glial cells and neurons. Compared with the control rats, the IOD/area of GnRH in the hypothalamus wassignificantly higher in rats treated with DEHP (*P*<0.05, Table 4). 

***Protein expression levels in the hypothalamus***

The protein levels of Kiss-1, GPR54, and GnRH in the hypothalamus were measured by Western blotting (Figure 6). The level of Kiss-1 was significantly higher in rats treated with DEHP compared with the control rats, while the protein level was higher in the rats treated with 500 mg/kg/d DEHP compared with the rats in the 250 and 1000 mg/kg/d DEHP groups (Figure 7A, *P*<0.05). The level of GPR54 was higher in the rats treated with 500 mg/kg/d DEHP compared with the rats in control, while the protein level of 1000 mg/kg/d dose group was significantly lower than 250 and 500 mg/kg/d groups (Figure 7B, *P*<0.05). The level of GnRH was higher in the rats treated with DEHP compared with the rats in control, while the protein level of 1000 mg/kg/d dose group was significantly lower than those of 250 and 500 mg/kg/d DEHP groups (Figure 7C, *P*<0.05).

## Discussion

DEHP is one of the most important plasticizers, through evaporation and migration into drinking water, food, air, and soil, and enters human and animal bodies through these materials (28). It can cause injuries to the brain, reproductive system, blood, liver, lung, kidney, and other organs (29). In recent years, girls are reaching puberty earlier. Adolescence is an important period in the development of the female reproductive system (30), and exposure to environmental endocrine disruptors before puberty can cause abnormal development (31). Initiation of puberty in the female is dependent on maturation and establishment of the hypothalamus-pituitary-ovarian axis, which affects the development of the female reproductive system by regulating the production and release of sex hormones. A previous study had shown that exposure to DEHP may lead to disruption of the estrogen synthesis pathway and disorders of the hypothalamus-pituitary-ovarian axis in adult female rats (32). DEHP also can increase the volume of the uterus in pubertal female rats, and raise the GnRHR mRNA and protein levels of the uterus (33). This study investigated the effects of DEHP on precocious puberty in female rats, and the results revealed the reproductive toxicity of DEHP on pubertal female rats. Thus according to these results, we will be able to provide a way for the prevention and or control of occurrence of precocious puberty in the girls.

Female rats have a stable estrous cycle and puberty, just like human beings. The vaginal opening time in female rats is usually used as a judgment of the onset of puberty and the establishment of the stable estrous cycle. The level of estrogen in female rats plays a decisive role in the vaginal opening time, and EEDs exposure may affect the vaginal opening time of female rats (34). The results of this study showed that the vaginal opening time of female rats treated with 1000 mg/kg/d DEHP was earlier than control and other groups, while the estrous cycle and anestrus of female rats treated with 500 and 1000 mg/kg/d DEHP were significantly longer than those of the control rats. These results indicated that DEHP exposure could shorten the vaginal opening time and prolong the estrous cycles of female rats.

GH and IGF-1 regulate the development and function of cells throughout the body (35). In the recent years, it is suggested that GH and IGF-1 could have effects on the central nervous system (CNS) (36). Information on DEHP exposure and hypothalamus function, as well as GH and IGF-1, in animal studies, is limited. And the effects of DEHP on GH and IGF-1 levels in puberty are unclear. Previous work had demonstrated a negative correlation between the serum IGF-1 levels among minors and the sum of urinary DEHP metabolite levels (37). In this study, we observed that the levels of IGF-1 in the hypothalamus of rats treated with 250 and 500 mg/kg/d DEHP were significantly lower than control rats, but the secretion of 1000 mg/kg/d DEHP was significantly higher than other groups. Meanwhile, there was no significant difference in the levels of IGF-1 expression in the serum. This might be explained by dramatically changed peripheral IGF-1 levels during fasting in humans and rodents, and the difference between central and peripheral signals. Our results also showed that GH levels in the hypothalamus and serum of pubertal female rats exposed to DEHP were significantly higher than those of the control group. We propose a mechanism by which Kiss-1 conveys reproductive and hormone status onto the somatotropic axis, resulting in GH release of the hypothalamus. It is suggested that DEHP might accelerate the growth and development of puberty in female rats by affecting the hormone levels of GH and IGF-1 in the hypothalamus and serum and induce precocious puberty.

Generally, hypothalamus-pituitary-ovarian axis regulates the secretion of FSH, LH, P, T, and E_2_. Surprisingly, in the present study, the serum levels of FSH, LH, and T were significantly increased after treatment with DEHP, and P was significantly reduced. These results verify that DEHP leads to the synthesis and secretion of P hormones due to the ovary. DEHP can competitively bind the endogenous estrogen receptor (ER), affect the transferring of hormone signals in the body, and lead to increased serum FSH and LH levels (38). The decrease in T hormone levels may be due to increased estrogen inhibition of T hormone synthesis.

The onset of puberty begins with increased GnRH in the hypothalamus, which initiates the hypothalamus-pituitary-gonadal axis (HPGA) (39). The activation of HPGA is affected by many factors, including neuroendocrine factors, such as γ-aminobutyric, kisspeptin, leptin, neuropeptide Y, and nitric oxide, and it is also concerned with exogenous estrogen or estrogen-like substances (40-43).

Studies have found that the Kisspeptin of the Kiss-1 gene in the hypothalamus is an important factor in HPGA activation, and that Kisspeptin regulates the synthesis and secretion of GnRH in the hypothalamus by binding and activating GPR54 on the GnRH neurons, which initiates and participates in the regulation of HPGA (44-46). Lower expression of Kiss-1 gene had no effect on the expression of the GnRH gene in the hypothalamus, but it decreased the concentration of GnRH in the blood, which indicates that Kisspeptin can affect the release of GnRH (47). The GnRH in mice with Kiss-1 gene knockout was lower, and the development of sexual organs was abnormal. After Kisspeptin treatment, the serum GnRH content was increased and the sexual function was improved (48). 

Kiss-1 and GPR54 can regulate reproductive and endocrine functions in the hypothalamus (49). In the development of rats, Kiss-1 and GPR54 mRNA levels in hypothalamus will increase and reach the peak in puberty. The Kiss-1 from the hypothalamic anteroventral periventricular nucleus (AVPV) stimulates GnRH secretion, whereas Kiss-1 in the arcuate nucleus (ARC) does the opposite (50-52). 

In this study, DEHP may reduce the mRNA levels of Kiss-1, but the expression of the Kiss-1 protein was not consistent with mRNA, which was observed in the group receiving DEHP with a significant increase. One hypothesis is that Kiss-1 neurons in the hypothalamus of female rats are sensitive to DEHP and can increase the efficiency of Kiss-1 mRNA transition, thus resulting in a decrease in Kiss-1 mRNA and an increase in protein content.

In addition, the mRNA levels of GPR54 and GnRH in the hypothalamus remained unchanged in the control and DEHP exposed groups. But the protein levels of GPR54 in 500 mg/kg/d DEHP dose group were higher than the control group, and the protein levels of GnRH treated with DEHP were also significantly higher than the control rats. It showed that DEHP could affect the post-translational regulation of GPR54 and GnRH in the hypothalamus of female rats, and the protein expression was stable and not easily fluctuated. The GPR54 protein in the hypothalamus of DEHP-treated rats was increased with the release of Kiss-1, which could stimulate the secretion of GnRH. Also, high dose of DEHP might inhibit the hypothalamic GPR54 and GnRH protein expression. It suggested that the levels of Kiss-1, GPR54, and GnRH in the hypothalamus might be an important target of DEHP and other exogenous endocrine disruptors.

DEHP exposure could affect the mRNA and protein expression levels of Kiss-1, GPR54, and GnRH in the hypothalamus in pubertal female rats, interfering with the endocrine regulation of the hypothalamus and affecting the development and reproductive function of the gonad.

## Conclusion

In summary, DEHP may have reproductive toxicity in pubertal female rats. DEHP exposure could shorten the time of the vaginal opening, and prolong the estrous cycle of pubertal female rats. DEHP exposure could influence the synthesis and secretion of GH, IGF-1, and sex hormones, and it also could affect the mRNA and protein expression levels of GnRH, Kiss-1, and GPR54 in the hypothalamus in pubertal female rats, interfering with the endocrine regulation of the hypothalamus and affecting the development and reproductive function of the gonad. In the present study, our data reveal that DEHP exposure may lead to hypothalamic imbalance in pubertal female rats and induce precocious puberty. 
